# Sensing Ability of Ferroelectric Oxide Nanowires Grown in Templates of Nanopores

**DOI:** 10.3390/ma13071777

**Published:** 2020-04-10

**Authors:** Mariya Aleksandrova, Tsvetozar Tsanev, Ashish Gupta, Ajaya Kumar Singh, Georgi Dobrikov, Valentin Videkov

**Affiliations:** 1 Department of Microelectronics, Technical University of Sofia, 1000 Sofia, Bulgaria; zartsanev@tu-sofia.bg (T.T.); georgi_hd@tu-sofia.bg (G.D.); videkov@tu-sofia.bg (V.V.); 2 National Centre for Flexible Electronics, Indian Institute of Technology Kanpur, Kanpur, Uttar Pradesh 208016, India; ash@iitk.ac.in; 3 Govt.V.Y.T.PG. Autonomous College, Durg, Chhattisgarh 491001, India; ajayaksingh_au@yahoo.co.in

**Keywords:** ferroelectric oxide, nanostructuring, anodic aluminum oxide, nanowires, piezoelectric response, pyroelectric response, multisensor device

## Abstract

Nanowires of ferroelectric potassium niobate were grown by filling nanoporous templates of both side opened anodic aluminum oxide (AAO) through radiofrequency vacuum sputtering for multisensor fabrication. The precise geometrical ordering of the AAO matrix led to well defined single axis oriented wire-shaped material inside the pores. The sensing abilities of the samples were studied and analyzed in terms of piezoelectric and pyroelectric response and the results were compared for different length of the nanopores (nanotubes)—1.3 µm, 6.3 µm and 10 µm. Based on scanning electronic microscopy, elemental and microstructural analyses, as well as electrical measurements at bending and heating, the overall sensing performance of the devices was estimated. It was found that the produced membrane type elements, consisting potassium niobate grown in AAO template exhibited excellent piezoelectric response due to the increased specific area as compared to non-structured films, and could be further enhanced with the nanowires length. The piezoelectric voltage increased linearly with 16 mV per micrometer of nanowire’s length. At the same time the pyroelectric voltage was found to be less sensitive to the nanowires length, changing its value at 400 nV/µm. This paper provides a simple and low-cost approach for nanostructuring ferroelectric oxides with multisensing application, and serves as a base for further optimization of template based nanostructured devices.

## 1. Introduction

Ferroelectric oxides are of great scientific interest as they are flexible in terms of properties such as tuning during deposition, technological compatibility with micro-/nanoelectromechanical systems (MEMS/NEMS) fabrication technology, and their applicability in energy harvesting or multisensor devices, due to their piezoelectric and pyroelectric response [1,2,3]. Ferroelectric nanowires could be incorporated into precise sensors, which have been found to exhibit better characteristics as compared to the bulk or thin film devices which are not nanostructured. Especially attractive are these elements when using them as small area, low power sensors in portable electronics due to the possibility of being autonomous [4,5]. 

There have been studies of ferroelectric oxides, such as potassium based oxides (sodium, niobium, lithium, tantalum, etc.) with potential applications in mechanical energy harvesting due to their favorable piezoelectric properties and durability at multiple activation. Their response can be precisely tuned by tailoring their composition and microstructure during crystal growth or film deposition [6,7,8,9]. There are also reports concerning the self-polarization ability of piezoelectric materials, which are grown as nanowires [10]. The assumption for that is the mechanism of nucleation and crystallization of the ferroelectric oxide—namely, from the inner walls first and then tending to fill the nanopore by reducing its diameter. The most studied nanowires used for sensing applications are ZnO ones [11,12]. Most often, a hydrothermal method is applied for their fabrication due to the low temperatures revealed during the process [13]. Although the above mentioned method provides vertical nanowires, they are not well aligned. Therefore, a template is needed in order to keep them array growing in a preliminary defined direction with repeatable size in terms of diameter and length [14]. 

Template-assisted growth is a mechanism for obtaining high resolution nanowires, which very often involve ink or solution as a filler, resulting in easy filling of the network with material in liquid or viscous form accompanied by the solvent evaporation process until a solid state nanowire is produced [15,16]. Physically, this is possible due to the difference in the free surface energy between the wetting pores and the filling solution (typically organic based) and the process is assisted by the good wetting ability of an anodic aluminum oxide (AAO) template. Die casting of molten zinc metal into the nanopores of AAO has been performed by vacuum depressurized of the mold and consequent annealing in an atmospheric environment for oxidation has been conducted for fabrication of well-ordered ZnO nanowires [17]. In this case, the piezoelectric current has been successfully measured as a function of the nanowires length. Chemical vapor deposition has been also practiced on an AAO substrate and it has been found that the growth mechanism is strongly dependent and limited by the vapor diffusion process into a solid template [18]. Synthesis of BaTiO_3_ nanowires via AAO has been demonstrated by filling the template with precursors of BaTiO_3_ and applying a vacuum [19]. There have been reports on AAO filling by means of piezoelectric Polyvinylidene fluoride (PVDF)-based polymer for poled nanowires growth [20,21]. Template-wetting growth of polymer nanowires has been demonstrated in [22], and showed preferable c-axis crystallization and polarization along the nanowires length, attributed to the mechanism of preferential nucleation from the pore walls toward the middle zone. Although the enhanced mechanical strength of such nanoelectromechanical systems, due to the polymer low Young’s modulus, the piezoelectric efficiency cannot be significantly increased after an increase in the surface-to-volume ratio, because of the relatively lower piezoelectric coefficients of the piezoelectric polymers, when compared with the piezoelectric oxides. Therefore, it is very important to find a way for effective filling of an AAO template with piezoelectric oxides, which are ceramic and are most often sputtered in vacuum. There is no sufficient information in the literature yet about the degree of filling of AAO nanotubes with sputtered piezoelectric oxide and how it affects the overall sensing performance. 

Pyroelectric properties also depend on the ferroelectric materials’ microstructures. It is already known that a single crystal potassium niobate possesses such ferroelectric behavior [23]. This is due to the orthorhombic symmetry of its lattice, making it very similar to barium titanate. The performance of the pyroelectric sensors is determined by the polarization distribution, which is responsible for the anisotropy of the pyroelectric effect in these domains. Typically, oxide coatings with thickness in the range of few hundred of nanometers to few micrometers are applied in the devices. However, their pyroelectric properties could be affected by the patterning or nanostructuring of the coating, in the same way the piezoelectric coatings behave. Although pyroelectric generators based on KNbO_3_ have been already investigated [24], by the authors’ knowledge, the possibility of using these nanowires templated by AAO for studying their pyroelectric sensing properties has not been demonstrated yet.

In our previous report, such behavior has been demonstrated and AAO with pores partially filled with sputtered KNbO_3_ at the inner walls has been shown [25]. Because the pores’ diameter is rather low, and the bottom side of the AAO template is not “opened”, but rather closed with the Al/AlO_2_ barrier layer, and so the pores cannot be evacuated from the filled air and the sputtering process partially fills the pores at their inner walls. In the present paper, we increase the diameter of the pores, optimize the growth conditions for the AAO template by dissipating the heat generated during anodization, and etch the bottom (closed) side to make a through hole. Then, suitable mounting of the samples in the vacuum deposition chamber provide conditions for plasma conduction and filling of the pores until high density nanowires from KNbO_3_ with well-defined lengths are formed. The aim is to assess the performance of such elements in terms of electromechanical and pyroelectric behavior with possible applications as sensors at different ferroelectric nanowire lengths. To the best of the authors’ knowledge, this is the first study of multisensing devices involving the combination of AAO templates and ferroelectric KNbO_3_ nanowire materials, combining the piezoelectric and pyroelectric principles of operation.

## 2. Materials and Methods 

**Figure 1:**
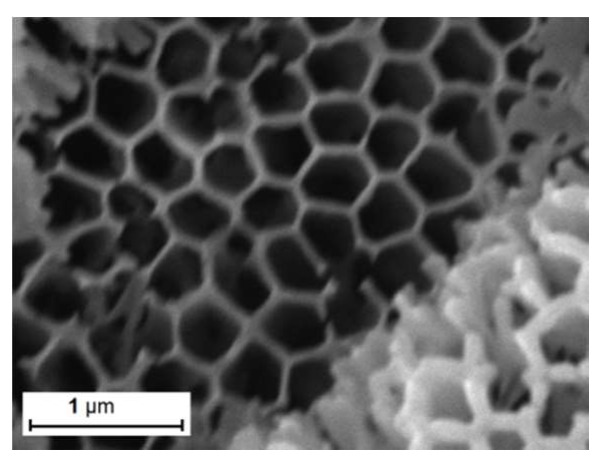
SEM image showing top view of uncoated sample with potassium niobate anodic aluminum oxide (AAO) pores.

Laboratory-made nanoporous AAO templates used in this work were produced following procedures described elsewhere [25], and had pores with an outer diameter of ~200 nm distributed over area of 0.9 cm^2^ (Figure 1). Opening of the backside of the “cuvette”-type tubes was done by dipping them in chromium-based etching solution. The AAO templates had different thicknesses of ~1.3 µm, 6.3 µm, and 10 µm, and a 40 nm wall separating the nanopores, forming in this way flexible membranes with porosity of approximately 60% with same pore size, but different surface-to-volume ratios due the different figures of merit of the nanowires’ lengths. In order to provide the filler for the template-assisted growth, potassium niobate ferroelectric ceramic target was sputtered in a vacuum. The established sputtering pressure of 2.10^−2^ Torr and sputtering voltage of 0.80 kV resulted in nanowires grown along the length of the template, filling the nanopores after both-sided sputtering of the piezoelectric material. Sensing structures ready for electrical measurements were produced by dip-coating with conducting polymer poly(3,4-ethylenedioxythiophene) polystyrene sulfonate (PEDOT:PSS) for better penetration in the pores that were not completely filled with KNbO_3_ along the full membrane thickness. After a drying procedure at 80 °C for 20 min, silver ink was applied on the PEDOT:PSS coating, providing serial electrical connection for all nanowires. The lack of electrical shortening at the opposite sides of the sensing element is evidence for the filling of the pores with piezoelectric material, rather than with conductive polymer and silver ink. Thin copper adhesive tapes were then attached to the samples in order to attach the measurement probes for the needs of membrane testing. They are also suitable for attachment to silicon wafers and to integrate elements on joint substrates with the electronics processing the sensor signal. 

For top view and cross-sectional monitoring of the produced nanopores and nanowires, scanning electron microscopes from LYRA I/FIB-SEM (TESCAN, Brno, Czechia) and Wl-103 A (Carl Zeiss, Jena, Germany) were used, respectively. X-ray diffraction (XRD diffractometer PW 1710, Philips, Eindhoven, The Netherlands) was used to determine the crystalline features of the nanowires after dissolving the AAO template. For the piezoelectric response detection system, periodic mechanical excitation was used, working in the 1-50 grams range to avoid sample destruction. Greater load causes membrane cracking. The mechanical stimulus was low frequency (50 Hz). The setup realized the cantilever-type measurement principle with a single fixed point. For the pyroelectric response, a Peltier heating-cooling module was used to set temperatures in the range of 2–58 °C. Generated AC and DC voltages were measured by an Agilent 34405A voltmeter (Agilent Technologies, Santa Clara, CA, USA). The produced piezoelectric and pyroelectric voltages were determined from sensor device outputs for 5 devices prepared in the conditions and the presented data were averaged with a relative error of ±2%.

## 3. Results and Discussion

**Figure 2:**
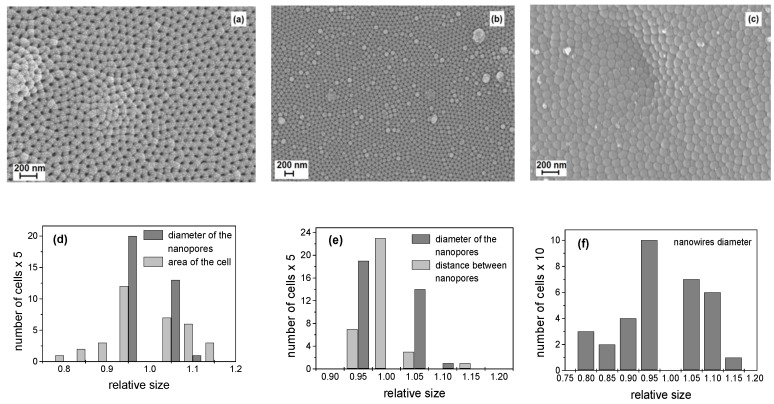
SEM images of the top view of the AAO pores with different filling degrees (different times of sputtering of KNbO_3_): (**a**) after 30 min of sputtering the pores are still open; (**b**) after 45 min the pores tend to be filled, but they are still partially open; (**c**) after 60 min of sputtering the pores are fully closed; (**d**) distribution of the nanopores diameters and cell areas; (**e**) distribution of the nanopores’ diameters and distance in between; (**f**) distribution of the nanowires’ diameters.

Figure 1 shows a top view SEM image of the nanopores’ geometry in the AAO template before filling with potassium niobate. Figure 2a–c shows the top view of the AAO after 30, 45, and 60 min of KNbO_3_ sputtering, which resulted in different degrees of gradually-filling nanopores—from fully opened (Figure 2a), through partially opened (Figure 2b) to fully closed (Figure 2c). A histogram of the relative nanopore diameters and cell areas (nanopores and separating walls), with respect to the theoretical values in nanometers [26], is shown in Figure 2d. It should be noted that most of the nanopores had a geometry close to the theoretical predictions, with 95% coincidence. Figure 2e presents a histogram of the nanopores’ diameters and the distance between the nanopores after partial filling with potassium niobate. The distribution is relative to the theoretical values when the assumption is for a 50% filing degree. It can be seen that the major portion of cells exactly fit in the geometry of the theoretical prediction. Figure 2f shows the relative distribution of the produced nanowires, which fully fill the nanopores. According to the histogram, most of the nanopores are characterized with 95% filling and those showing relative diameter greater than one are due to irregular formations, or particle heaping, which hinder correct recognition of the surface objects by the software. 

**Figure 3:**
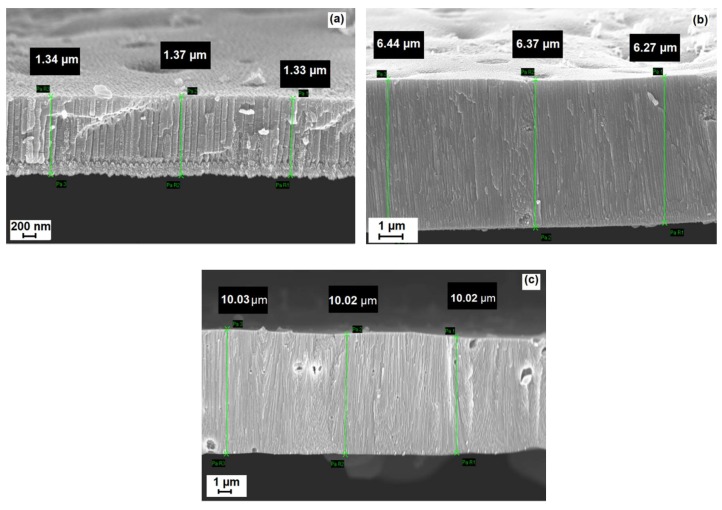
Cross section of nanowires with different length: (**a**) 1.3 µm; (**b**) 6.3 µm; (**c**) 10 µm.

A cross-sectional view of the nanowires grown in the pores and having three different lengths are shown in Figure 3a–c. The resulting nanowires were densely arrayed, and good vertical alignment was found. Some defects, due to the membrane break, appeared in the images. The nanowires’ diameters of approximately 150 nm were rather identical in all types of samples, no matter the length. This is possible because template-assisted growth supports good regularity.

**Figure 4:**
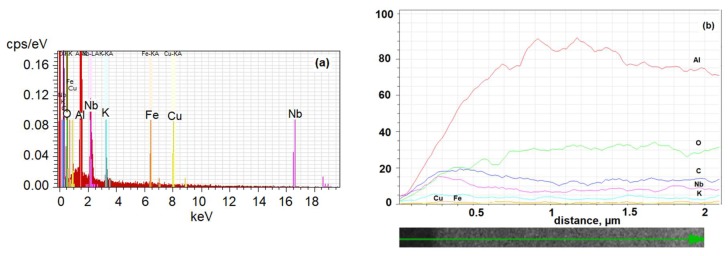
EDS analysis of AAO nanopores filled by KNbO_3_: (**a**) elements presented in the nanopores; (**b**) distribution of the elements inside the AAO nanotubes along their length.

Elemental analysis energy-dispersive spectroscopy (EDS) was conducted in points that were selected from the middle part of the break (Figure 4a,b) and confirmed that potassium niobate was present in the pores in a ratio close to that defined by the manufacturer in the material specification [27]. As can be seen from the presence of specific elements (K, Nb) along the nanotube length, nanowires with uniform distribution of these elements were produced. Some by-products of the etching procedure captured in the AAO pores gave negligible additional signals in the EDS spectrum (e.g. Fe, Cu), which can be ignored.

**Figure 5:**
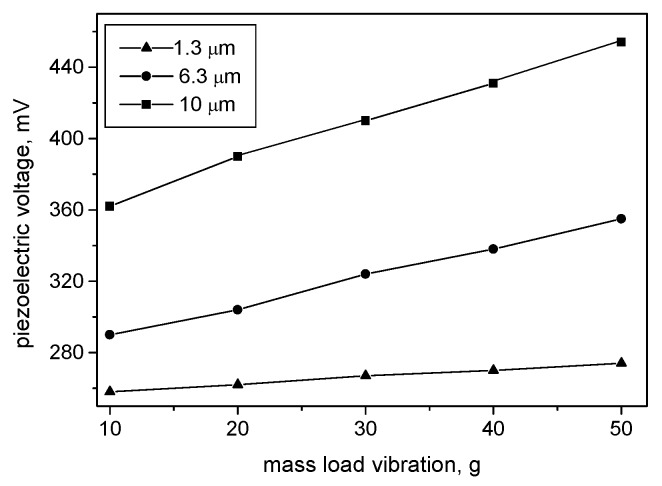
RMS voltage of AAO template-based sensor as a function of the mass load at 50 Hz.

Figure 5 shows a dependence of the root mean square (RMS) voltage, produced from theKNbO_3_-filled AAO membrane as a function of the mass load, demonstrating the device’s sensitivity at different lengths of the nanowires, when a low frequency vibration of 50 Hz loads the structure. It was found that the produced voltage is at a maximum around this frequency and the slightly decreases, or deviates, when the vibration frequency respectively decreases, or increases. RMS values were recorded, because the signal processing integrated circuits that further use the signals for amplification and filtration are not designed as peak detectors and work with RMS values.

As can be seen from Figure 5, the sensor exhibited a response with excellent linearity with respect to the mass loading in all three cases. Increasing the length of the piezoelectric nanowire resulted in increasing the sensitivity in the studied range of load, as can be noted from the slopes of the characteristics. This is in good agreement with theoretical predictions [28], as well as with the previously reported results demonstrating that the piezoelectric charge of a nanowire is proportionally induced to the length of the nanowire [29]. It was proven that at higher nanowire lengths, the flexibility of the nanostructured element is greater, so it is easier to achieve the generation of a higher output voltage, because of the greater deflection of the nanowires [30]. Structures with nanowire lengths of 1.3 µm showed 0.4 mV/g, nanowire lengths of 6.3 µm showed 1.7 mV/g, and nanowires lengths of 10 µm showed 2.3 mV/g (aspect ratio varies between 5 and 40). 

**Figure 6:**
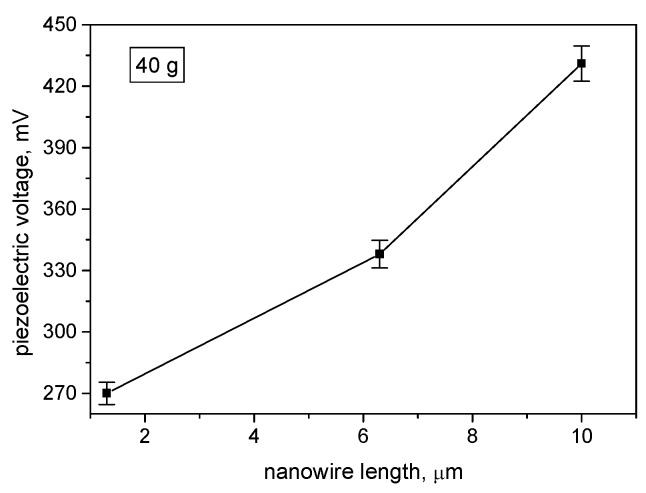
Piezoelectric voltage as a function of the piezoelectric nanowire length at constant mass load.

**Figure 7:**
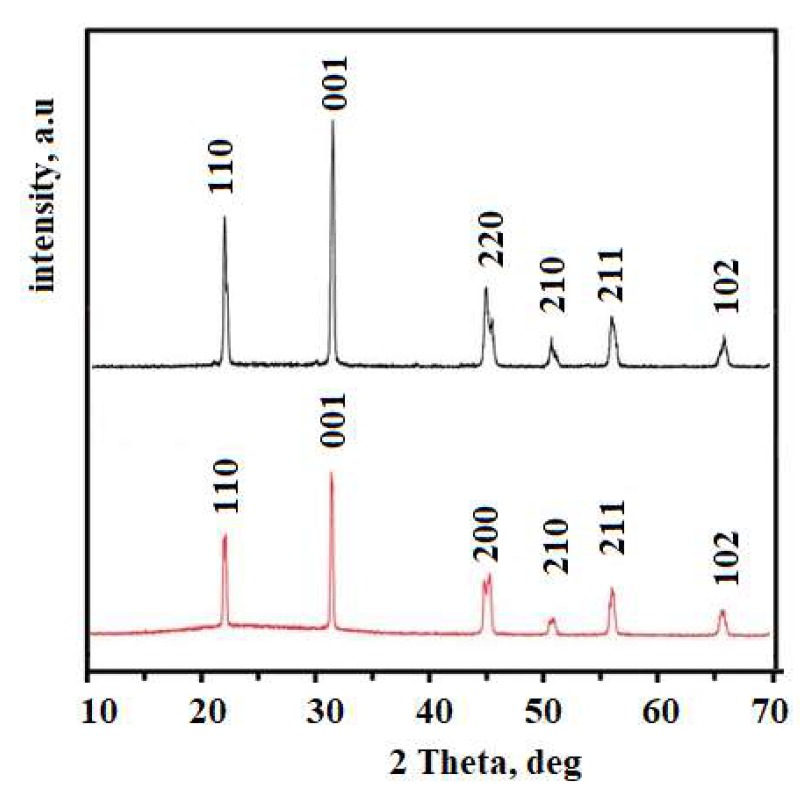
XRD patterns of KNbO_3_ nanowires with length 1.3 µm and 10 µm produced by AAO template.

Figure 6 depicts the piezoelectric voltage dependence on the nanowire length. The voltage was higher for the longer nanowires. This could be ascribed to the greater volume of the piezoelectric material, or to the higher stress that longer nanowires experience. In order to better clarify this relation, XRD measurements were conducted and data about the shortest and the longest nanowires were presented for comparison (Figure 7). At diffraction angle of 31°, a strong diffraction peak was observed for all nanowire lengths. The peak intensity seemed to be proportional to the length of the nanowires. Analysis of the XRD diagrams and reference to the JCPDS (Joint Committee on Powder Diffraction Standards) database revealed that the peak value was generated by the KNbO_3_ of the (001) side. Therefore, the produced nanowires are characterized by c-axis-oriented growth, which is preferable for fabrication of transducers with high piezoelectric response. The patterns revealed an orthorhombic structure of the KNbO_3_, which suggests its ferroelectric behavior [31].

Comparison with non-structured potassium niobate 410 nm thin films showed that at a mass loading of 20 g, the produced piezoelectric voltage was 483 mV [32]. Taking into account that the collected charge is dependent on the electrode coverage area, and the reported active area is 4 cm^2^, then it could be noted that piezoelectric nanowires with a length of 10 µm could produce 390 mV from an active area 4.5 times smaller at the same mass loading. The error bar is small due to the good repeatability in the regularity of the AAO template and good reproducibility of the vacuum-deposited KNbO_3_, also suggesting good control over the nanopores filling process. Deviations that occurred can be ascribed to possible defects due to lack of completeness of some nanopores, resulting in partial filling or causing undefined geometry of the grown material. The reason could be the purity of the raw aluminum (99.8%). 

**Figure 8:**
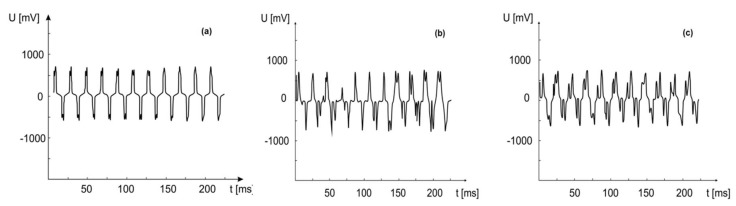
Generated voltage from element AAO/KNbO_3_ with different piezoelectric nanowires length at multiple press-release excitations: (**a**) 1.3 µm; (**b**) 6.3 µm; (**c**) 10 µm.

Figure 8a–c represent the generated piezoelectric voltage at press-release loading with a frequency of 50 Hz, produced from elements with nanowire lengths of 1.3 µm, 6.3 µm, and 10 µm. Strong symmetry of the signal was observed with respect to the zero volt axis, which is evidence for a piezoelectric response with clear dependence on the nanowire length. 

**Figure 9:**
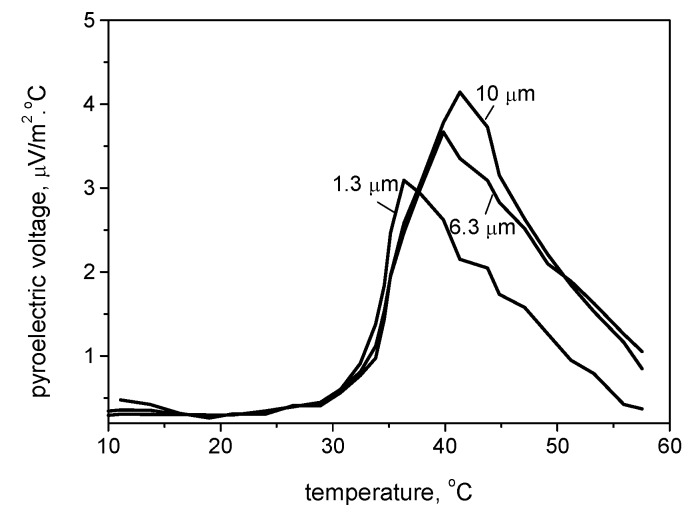
Pyroelectric coefficient of the KNbO_3_ nanowires as functions of their length.

The envelopes of the pyroelectric coefficient curves depend on the weakness of the input stimulus and on the nanowires length as compared to the piezoelectric responses dependence. These relations are shown in Figure 9, where the pyroelectric voltage was measured in the temperature range of 2–58 °C for the three nanowire lengths of 1.3, 6.3, and 10 µm. It could be noted that in contrast to the piezoelectric voltage, which increase gradually and linearly with the mass load, the pyroelectric voltage follows a fast exponential increase with the thermal load. 

The curves are characterized by a shape typical for pyroelectric materials, with one maximum that depends on the coating microstructure. The pyroelectric voltage, which is a link between the polarization degree and the temperature change, is higher at the longest KNbO_3_ nanowires of 10 µm, which could be a reason for a greater voltage drop. It should be also noted that the maximum of the curve is shifted toward the higher temperature (~42 °C) as compared to the shortest nanowire, where the maximum is situated at 36 °C. This may be ascribed to the higher nanowire polarizable volume at the longer length [33].

The resistivity R of the nanowires can be determined by using the relation (1):

(1) $$R=\frac{4\rho L}{\pi d^{2}},$$

where *L* is the nanowire length, *d* is the nanowire diameter, and ρ is the specific resistance of the material, no matter its band gap [34,35]. According to this, longer nanowires exhibit higher resistance. Since the nanowire diameter is in the range of 100–120 nm, any changes in the electrical resistance along the nanowire length will influence the polarization ability significantly. As the pyroelectricity is defined as a change of the material’s spontaneous polarization due to a change in temperature, then it is expected that the polarization will be different for the samples with different nanowire lengths, due to the different polarizable volumes. It seems that the pyroelectric voltage gradually disappears at certain threshold temperatures, which depends on the nanowire length. Similar effects have been previously observed for ferroelectric oxide nanowires, and the pyroelectric voltage decrease was ascribed to electrical losses due to leakage currents, which in this case appear at higher temperatures for the longer nanowires because of their higher resistivity [36]. Although the observed effect requires further investigation, this could explain the trend for pyroelectric voltage reduction, which is nanowire length-dependent. 

## 4. Conclusions

The successful filling of AAO templates by sputtered potassium niobate for nanowire fabrication was demonstrated. Aligned nanowires are of great advantages in terms of piezoelectric sensor application, because they increase sensitivity to mechanical impacts. The nanowires showed preferential c-axis-oriented growth and orthorhombic structures, proving their ferroelectric nature. The structures showed a pyroelectric response, so in this work the possibility for using the prepared structures for a multi-sensor device, combining piezo- and pyroelectric behavior, was presented. The nanostructure affects the piezoelectric response to a great extent, while the pyroelectric response seemed less sensitive to the length of the nanowires. The prepared multi-sensor device is able to detect linear mass loading ranging from 10 to 50 g with a frequency of 50 Hz. A stable pyroelectric voltage in the temperature range of 10–40 °C was observed, but the pyroelectric properties require further investigation. 

Future work will be related to determining the long-term stability of the membranes, extending the mass and thermal loading ability of the elements, as well as separating the pairs of electrodes responsible for measurements of the AC and DC output signals as a result of the piezoelectric and pyroelectric effects.
